# Conversations with glaucoma patients

**Published:** 2012

**Authors:** Hannah Faal

**Affiliations:** Chairperson: Africa Vision Research Institute, Durban, South Africa.

**Figure:**
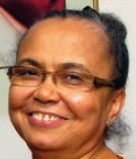
Hannah Faal

With a chronic eye condition such as glaucoma, patients and their carers also become ‘eye care workers’. Without their acceptance of surgery or active participation in their own medical treatment, you will not be able to preserve their sight!

Assuming that you have taken into consideration the patient's personal and financial circumstances (see pages 71–72), other reasons that patients may not follow medical advice include:

- having a poor understanding of the benefits of treatment
- the occurrence of side effects that had not been discussed with the patient
- poor communication or lack of trust between the patient and his or her health care provider.

So it is important to make time to talk and to listen to patients and their carers **as often as needed** and to respond honestly to any fears and concerns.

‘ICE’ is an easy acronym to help you remember the key areas to cover when talking with your patients. It stands for ideas, concerns, and expectations:

- **Ideas (beliefs)**: what are patients’ ideas about their condition? Knowing these means you can build on what they know already, and/or (gently) correct any incorrect or harmful ideas and beliefs they may have. Ask questions such as: ‘What do you think might be happening?’
- **Concerns**: what are their concerns about the condition, signs, symptoms, treatments, etc. Know what they are and address each one.
- **Expectations**: what are patients’ expectations of staff, or of that particular visit, admission to hospital, or operation?

The lists below^1^ give suggestions for what patients and their carers may wish to know. If your clinic is very busy, it may be a good idea to train a nurse counsellor who will be able to spend time talking with patients and carers about the different concerns and questions they may have.

**FROM THE FIELD**

**How I talk to patients with primary open-angle glaucoma**

**Figure:**
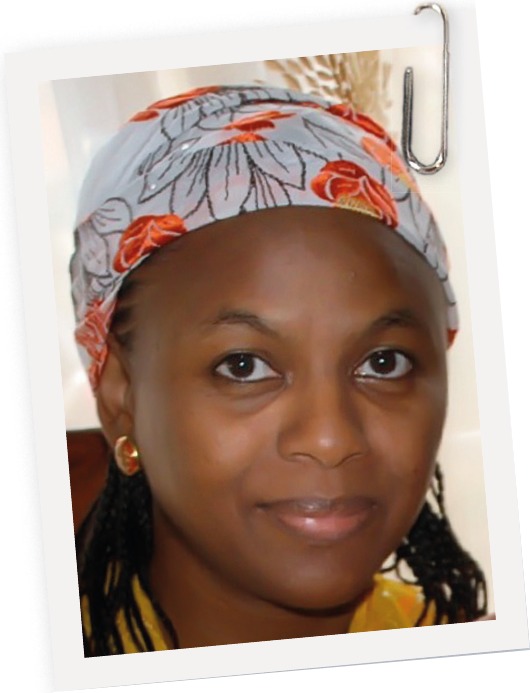

**Fatima Kyari** is an ophthalmologist in the Department of Ophthalmology, University of Abuja, Nigeria. She is studying for a PhD at the International Centre for Eye Health, London School of Hygiene and Tropical Medicine, London UK.

Patients, especially those who are newly diagnosed with primary open-angle glaucome (POAG), have many questions. Consultation times are short, so it can be useful to give patients some generic information about glaucoma in written form.

Even within the short consultation time, however, my usual practice with newly-diagnosed patients is to explain:

1. Basic information about glaucoma; i.e. the pressure within the eye is too high, which damages the nerve at the back of the eye without causing any pain. They will experience a gradual loss of vision if no treatment is given.
2. How we can stop or delay vision loss – a brief explanation of treatment. options: medicines, surgery, laser
3. The patient's visual prognosis in terms of treatment and adherence to medications, etc. (see above).

Then I ask the counsellor, if there is one, to explain further.

I would recommend that all busy glaucoma clinics employ a glaucoma counsellor.

Where I work, it seems that most patients would rather hear the initial explanation from the doctor. They are then better able to understand and relate to any further explanation from someone who has more time to counsel or motivate them.

## General

Talk to patients and carers about:

- their specific condition, its lifelong implications, and the likelihood they will keep their sight, provided they accept surgery and/or adhere to treatment
- the reasons you decided to offer them this particular type of treatment, as well as the risks and benefits
- opportunities for referral: community-based rehabilitation, patient groups, or low vision clinics
- requirements to drive legally.

## Medical treatment

Talk to patients and carers about:

- the importance of their role in their own treatment
- how to apply eye drops
- possible side effects
- what the treatment will involve, how it will help them, and what sort of improvements they might expect
- what they should do if they have a bad reaction to the eye drops, forget to use the eye drops, or use too much.

## Surgical treatment

Talk to patients and carers about:

- how long they will be in the hospital
- how much it will cost
- whether they will have any pain during and/or after the opration
- how they can expect their vision to change after the operation
- where they have to go, what day and time, and what they should expect. If possible, call the clinic and make the appointment while the patient is present.

## Monitoring/follow-up

Talk to patients and carers about:

- how often they have to come back to hospital
- which tests they will need, and what will happen at each visit
- how to recognise when their vision is getting worse, and what they should do.

## IMPORTANT

- Glaucoma patients should check their own fields by closing one eye at a time and come back if they notice any changes
- Patients should return to the clinic if their spectacle prescription changes very rapidly (e.g. every six months)
- Encourage all patients with glaucoma to bring their first-degree relatives for an eye examination.
